# Silencing MED1 Sensitizes Breast Cancer Cells to Pure Anti-Estrogen Fulvestrant In Vitro and In Vivo

**DOI:** 10.1371/journal.pone.0070641

**Published:** 2013-07-30

**Authors:** Lijiang Zhang, Jiajun Cui, Marissa Leonard, Kenneth Nephew, Yongquan Li, Xiaoting Zhang

**Affiliations:** 1 Department of Cancer Biology, University of Cincinnati College of Medicine, Cincinnati, Ohio, United States of America; 2 Institute of Biochemistry, College of Life Science, Zhejiang University, Hangzhou City, China; 3 Center of Safety Evaluation, Zhejiang Academy of Medical Sciences, Hangzhou City, China; 4 Department of Medical Sciences, Indiana University School of Medicine, Bloomington, Indiana, United States of America; Florida International University, United States of America

## Abstract

Pure anti-estrogen fulvestrant has been shown to be a promising ER antagonist for locally advanced and metastatic breast cancer. Unfortunately, a significant proportion of patients developed resistance to this type of endocrine therapy but the molecular mechanisms governing cellular responsiveness to this agent remain poorly understood. Here, we’ve reported that knockdown of estrogen receptor coactivator MED1 sensitized fulvestrant resistance breast cancer cells to fulvestrant treatment. We found that MED1 knockdown further promoted cell cycle arrest induced by fulvestrant. Using an orthotopic xenograft mouse model, we found that knockdown of MED1 significantly reduced tumor growth in mice. Importantly, knockdown of MED1 further potentiated tumor growth inhibition by fulvestrant. Mechanistic studies indicated that combination of fulvestrant treatment and MED1 knockdown is able to cooperatively inhibit the expression of ER target genes. Chromatin immunoprecipitation experiments further supported a role for MED1 in regulating the recruitment of RNA polymerase II and transcriptional corepressor HDAC1 on endogenous ER target gene promoter in the presence of fulvestrant. These results demonstrate a role for MED1 in mediating resistance to the pure anti-estrogen fulvestrant both *in vitro* and *in vivo*.

## Introduction

The estrogen receptor alpha (ERα) is a ligand-activated nuclear receptor that regulates the transcription of estrogen-responsive genes in many target cells. Upon binding estrogen, ERα is able to bind to the estrogen responsive element (ERE) and activate the expression of diverse target genes to enhance cell proliferation and tumorigenesis [1–3]. About 70% of human breast cancers express ERα and endocrine-based therapies aimed at blocking ERα action have been widely employed for breast cancer therapy [4]. Most prevalent substance classes for endocrine therapy include selective estrogen receptor modulators (SERMs, e.g. tamoxifen), selective estrogen receptor downregulators (SERD, e.g. fulvestrant) and aromatase inhibitors (AI, e.g. anastrozole) [4,5]. However, resistance to these endocrine therapies often occurs, which has become an increasingly pressing issue in current breast cancer therapy [6–10].

Fulvestrant has been used as both first- and second-line therapy for locally advanced and metastatic breast cancer [11]. Unlike the selective estrogen receptor modulator tamoxifen, fulvestrant has no known estrogen agonist activity and is considered a pure antiestrogen [12,13]. It competitively binds to ER with high affinity and induces a conformational rearrangement that leads to accelerated degradation of ER protein [14,15]. Considerable data have demonstrated the efficacy of fulvestrant in postmenopausal women with ER-positive advanced breast cancer, particularly in patients who have developed resistance to prior endocrine therapies such as tamoxifen or aromatase inhibitors [16]. Unfortunately, a significant proportion of patients developed progressive disease under fulvestrant treatment, which has become a critical issue for the endocrine therapy [17]. Unlike tamoxifen resistance, for which considerable data have demonstrated the mechanism of resistance in the past decades, the mechanism of the responsiveness of breast cancer cells to fulvestrant and the development of resistance remains understudied [9,18]. Recent reports have suggested the association of fulvestrant resistance with NF-κB signaling pathway, autophagy, cytokine receptor, microRNA expression and activation of growth-stimulatory pathways such as receptor tyrosine kinases of the epidermal growth factor receptor (EGFR) family and the insulin-like growth factor receptor (IGF-1R), etc [19–28].

Mediator Subunit 1 (MED1; also known as PBP or TRAP220) has recently been established as a key ERα coactivator both *in vitro* and *in vivo* [29–31]. MED1 is required for estrogen receptor-dependent reporter and endogenous gene expression and estrogen-dependent breast cancer cell growth [30,32–35]. *In vivo* animal model studies indicated that MED1 is required for estrogen receptor functions in pubertal mammary gland development and luminal progenitor/stem cell differentiation [31]. MED1 has been reported to be amplified and overexpressed in over half of human breast cancers [32,36–38]. Significantly, recent studies found that MED1 expression highly correlates with poor clinical outcome of breast cancer patients treated with endocrine therapy [39]. We have recently demonstrated that MED1 plays a critical role in tamoxifen resistance *in vitro* through direct crosstalk with the receptor tyrosine kinase HER2 signaling pathway [40].

Here, we report that MED1 plays an important role in mediating fulvestrant resistance not only *in vitro* and but also *in vivo*. We found that knockdown of MED1 significantly sensitized and further promoted fulvestrant-induced cell cycle arrest of fulvestrant resistance breast cancer cells. The *in vivo* roles of MED1 in breast cancer cell growth and fulvestrant resistance were further determined using orthotopic xenograft mouse models. Finally, the mechanisms underlying MED1 functions in fulvestrant resistance were also investigated.

## Results

### Knockdown of MED1 sensitizes breast cancer cells to fulvestrant treatment *in vitro*

**Figure 1:**
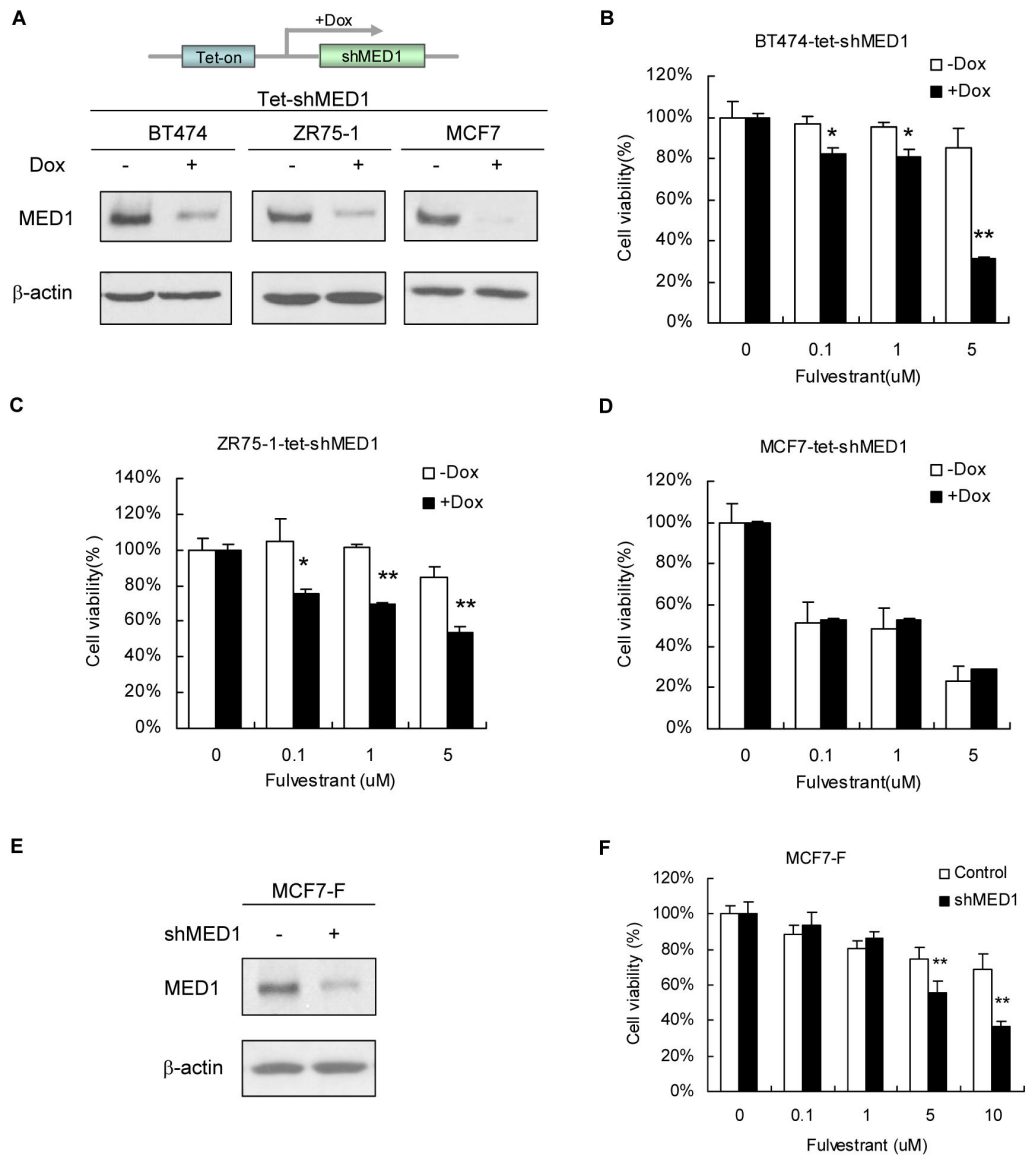
Knockdown of MED1 sensitizes breast cancer cells to fulvestrant treatment. (**A**) BT474-tet-shMED1, ZR75-1-tet-shMED1 and MCF-7-tet-shMED1 cells were treated with vehicle (-Dox) or doxycycline (+Dox) and subjected to western blot analyses using indicated antibodies. (**B**) (**C**) (**D**) MTT assays measuring cell proliferation of cells in (A) after control and fulvestrant treatments for 7 days. Relative cell numbers of both vehicle (-Dox) and doxycycline (+Dox) were normalized to that of their respective fulvestrant untreated control (set as 1). (**E**) Fulvestrant-resistant MCF7-F cells were infected with lentivirus expressing control scramble or MED1 shRNA and blotted for MED1 expression using β-actin levels as a control. (**F**) The cells in (E) were subjected to MTT assay after treatment with indicated concentration of fulvestrant. Relative cell numbers of both vehicle (-Dox) and doxycycline (+Dox) were normalized to that of their respective fulvestrant untreated control (set as 1). (n=4. *: P< 0.05; **: P< 0.01).

To investigate whether MED1 affects the sensitivity of breast cancer cells to fulvestrant, we generated BT474, ZR75-1 and MCF7 cells that stably express Tet-inducible MED1 shRNA. As shown in Figure 1 A, the expressions of MED1 in all these Tet-inducible cells (BT474-tet-shMED1, ZR75-1-tet-shMED1 and MCF7-tet-shMED1) were significantly down-regulated after Dox treatment when compared with vehicle control (-Dox) treatment. Consistent with previous studies [21,41], we found that BT474 and ZR75-1 cells were resistant to fulvestrant treatment. However, knockdown of MED1 significantly sensitized BT474 and ZR75-1 cells to fulvestrant treatment (Figure 1B, C). In contrast, the fulvestrant sensitivity of MCF7 cells that were already highly sensitive to fulvestrant was not significantly affected by Dox-induced knockdown of MED1 (Figure 1D). In addition, we performed MTT assays as above using control Tet-shGFP cells, as well as additional BT474-tet-shMED1, ZR75-1-tet-shMED1 and MCF7-tet-shMED1 clones generated. As shown in Figure S1, we obtained very similar results that further support our above conclusions. Moreover, we used another fulvestrant-resistant cell line MCF7-F to further investigate the effect of MED1 on fulvestrant sensitivity. We found that knockdown of MED1 by MED1 shRNA but not control scramble shRNA significantly sensitized the otherwise fulvestrant-resistant MCF7-F cells to fulvestrant treatment (Figure 1E and F). Together, these data strongly support a role of MED1 in mediating fulvestrant resistance in these breast cancer cells *in vitro.*

### Silencing MED1 promotes fulvestrant-induced cell cycle arrest in BT474 and ZR75-1 cells

**Figure 2:**
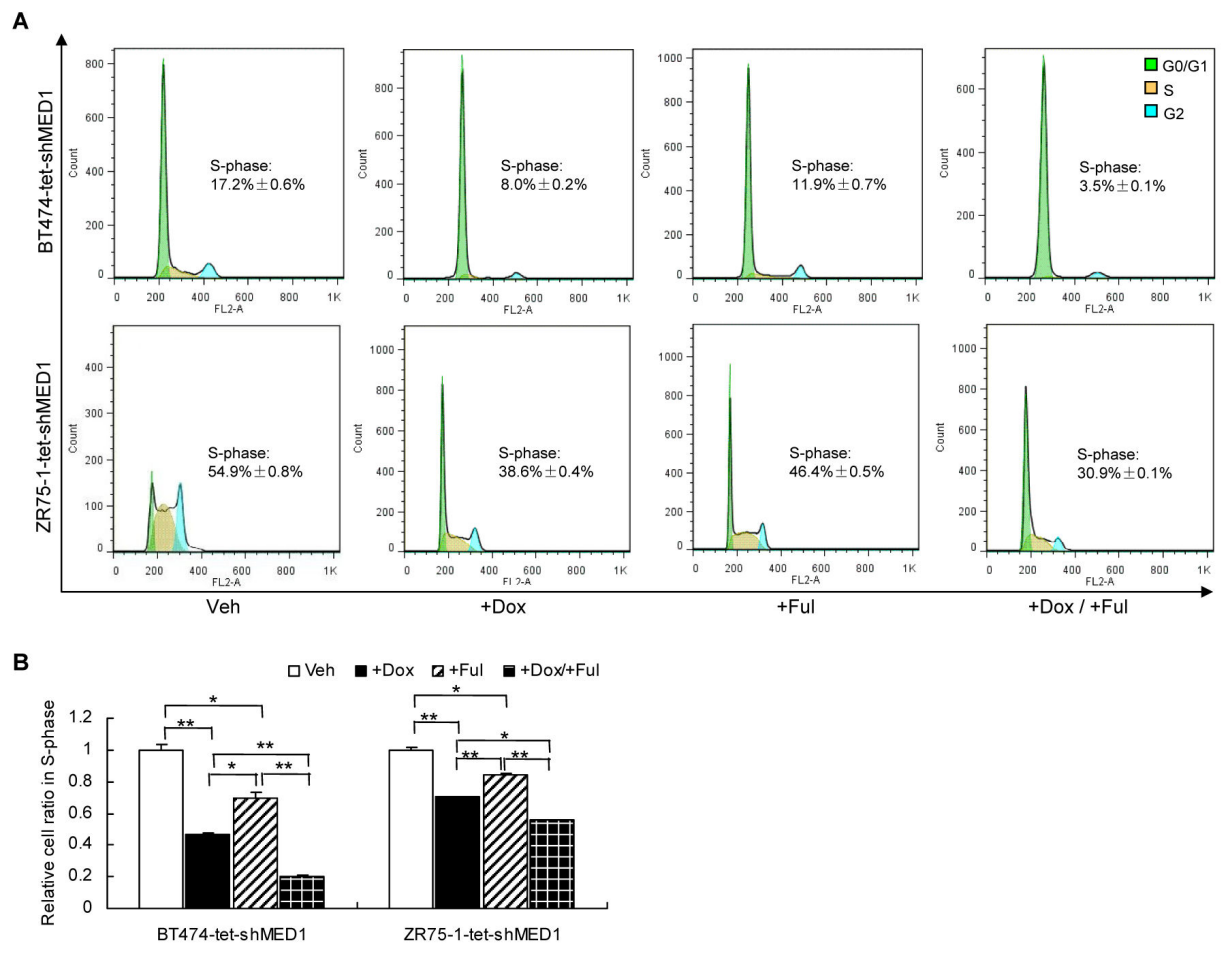
MED1 Knockdown promotes fulvestrant-induced cell cycle arrest. (**A**) BT474-tet-shMED1 and ZR75-1-tet-shMED1 cells were treated with vehicle (Veh) or doxycycline or/and fulvestrant (+Ful, 5µM) and harvested for flow cytometry analysis. The representative histograms of cell cycle distribution were shown with the percentages of S-phase cells presented. (**B**) The bar graph of the percentages of S-phase cells in above treatment groups after normalizing to that of the vehicle control treatment. (n=3. *: P< 0.05; **: P< 0.01).

Fulvestrant is known to induce cell cycle arrest during the G0/G1 to S transition in ER-positive breast cancer cells [21,42]. To investigate whether silencing MED1 could have an effect on cell cycle arrest induced by fulvestrant, we analyzed the cell cycle distribution in BT474 and ZR75-1 inducible cells after treatment with Dox and/or fulvestrant. We found that either fulvestrant treatment or MED1 knockdown reduces the percentage of S-phase cells in BT474 and ZR75-1 cells (Figure 2A and B). Importantly, combined treatment of both fulvestrant and MED1 knockdown further promoted cell cycle arrest in both of these cells(Figure 2A and B). These data indicate that down-regulation of MED1 can further potentiate the effect of fulvestrant on blocking S-phase entry.

### MED1 knockdown further potentiates fulvestrant-mediated inhibition of ER target gene expression

**Figure 3:**
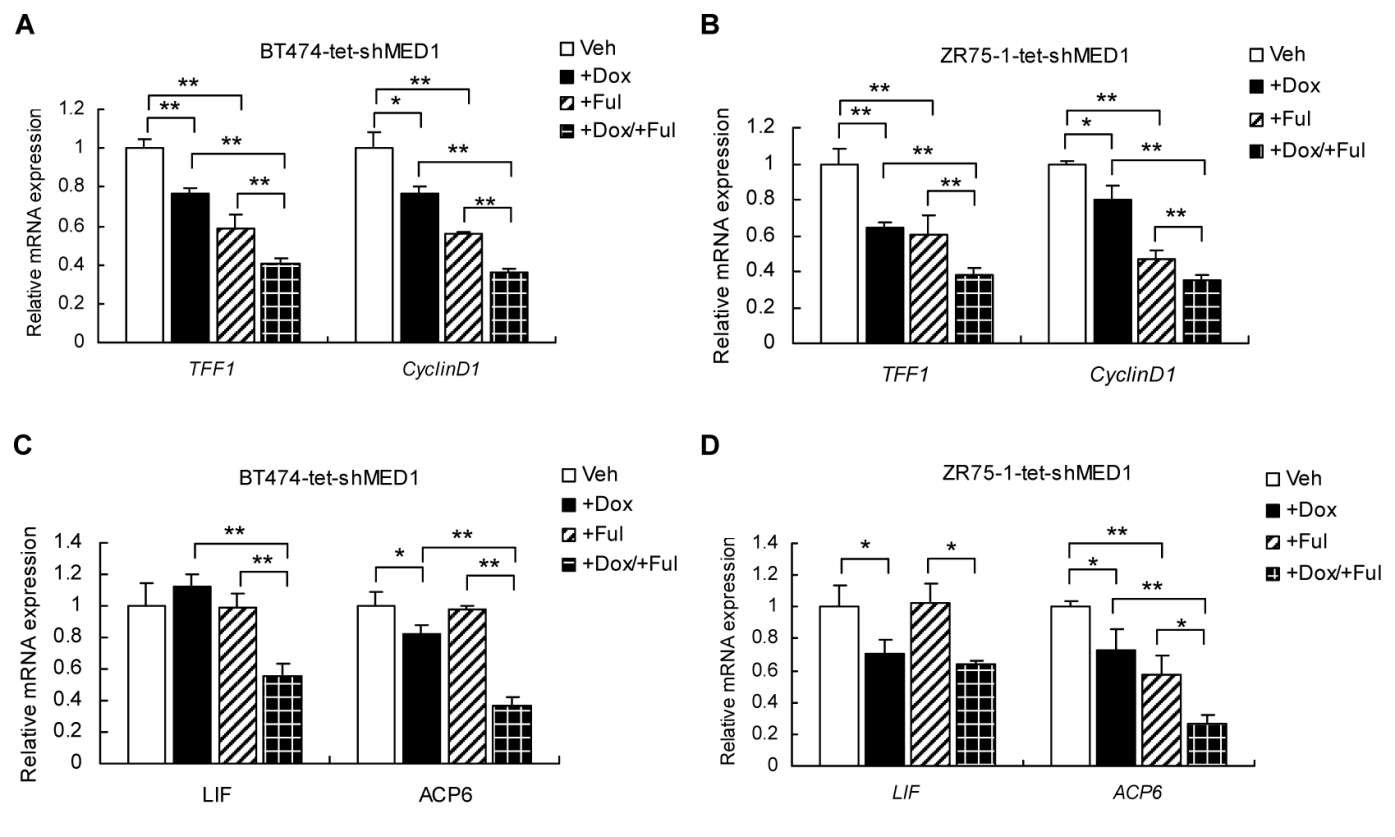
Silencing MED1 promotes down-regulation of ERα target genes induced by fulvestrant. (**A**) and (**B**) BT474-tet-shMED1 and ZR75-1-tet-shMED1 cells were first treated with vehicle (Veh) or doxycycline or/and fulvestrant (+Ful). Total RNA was then extracted, and the expression of E2-induced ERα target genes *TFF1* and *Cyclin D1* (A) and EGF-induced ERα target genes *ACP6* and *LIF* (B) was measured by real-time RT-PCR after normalization to that of *GAPDH*. (n=3. *: P< 0.05; **: P< 0.01).

To examine the molecular mechanisms underlying the above observed phenotype, we investigated the effect of MED1 knockdown on the expression of well-known ERα target genes (*TFF1*, *Cyclin D1*) in the presence and absence of fulvestrant treatments. As expected, fulvestrant treatment or MED1 knockdown alone is able to significantly down-regulate the mRNA expression of *TFF1* and *Cyclin D1* in both BT474-tet-shMED1 and ZR75-1-tet-shMED1 cells (Figure 3A and B). Importantly, combined fulvestrant treatment and MED1 knockdown further decreased the mRNA level of these genes when compared with treatment with either alone. Most recently, a genome-wide ERα-cistrome analysis has revealed that growth factors such as EGF can stimulate the binding of ERα to a distinct group of target genes (e.g. *ACP6* and *LIF*) [43]. Since we have previously shown that MED1 is also required for the expression of these genes [40], we decided to further examine whether MED1 also plays a role in the expression of this type of ER target genes in the presence of fulvestrant. Interestingly, we found that combining fulvestrant treatment with MED1 knockdown significantly downregulated the expression of both *ACP6* and *LIF* genes when compared to that of fulvestrant or MED1 knockdown alone treatment groups (Figure 3C and D). Moreover, since the fulvestrant-resistant MCF7-F cells were stably integrated with reporter ERE-TFF1-Luc to monitor the transcriptional activity of ER [24], we also examined the expression of this reporter activity. Consistent with above results, we found that knockdown of MED1 can further inhibit the ER transcriptional activity in the presence of fulvestrant (Figure S2). Together, these data indicate that MED1 knockdown can further potentiate fulvestrant-mediated inhibition of ER target gene expression.

### Silencing MED1 affects fulvestrant-modulated recruitment of RNA pol II and transcriptional corepressor HDAC1 to the *TFF1* promoter

**Figure 4:**
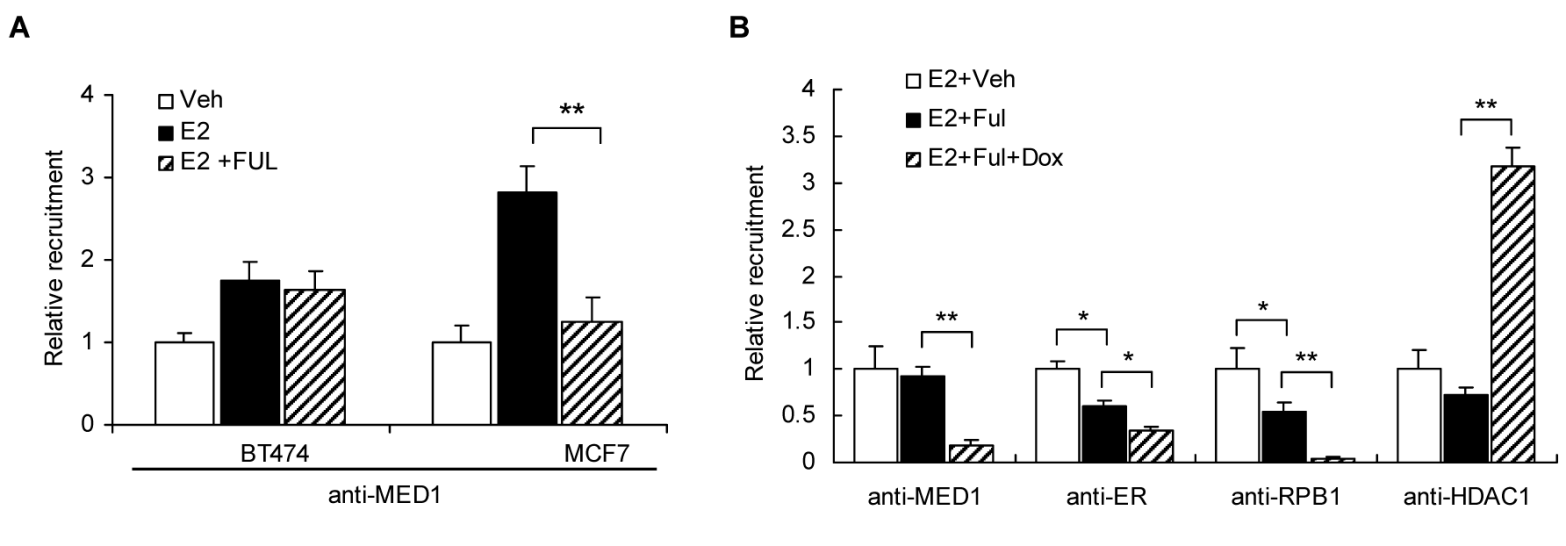
MED1 affects the recruitment of RNA pol II and transcriptional corepressor HDAC1 to *TFF1* promoter in the presence of fulvestrant. (**A**) MCF-7 and BT474 cells were pretreated with fulvestrant (Ful) or vehicle (Veh) for 6h, then followed by treatment with 17β-estradiol (E2, 100nM) or vehicle for 45min. ChIP assays were performed to detect the recruitment of MED1 on *TFF1* promoter using antibodies against MED1. (**B**) BT474-tet-shMED1 cells were pre-incubated with vehicle or doxycycline (Dox) for 7 days, followed by treatments as in (A). ChIP assays were performed to detect the recruitment of MED1, ERα, RPB1 and HDAC1 to *TFF1* promoter using respective antibodies. (n=3. *: P< 0.05; **: P< 0.01).

To further examine the molecular mechanisms underlying combined effects of fulvestrant treatment and MED1 knockdown on the expression of ER target genes, we performed chromatin immunoprecipitation (ChIP) assays. We found that fulvestrant treatment is able to inhibit the recruitment of MED1 on the *TFF1* promoter in fulvestrant-sensitive MCF-7 cells as expected. However, interestingly, the recruitment of MED1 to the *TFF1* promoter is not affected by fulvestrant in fulvestrant-resistant BT474 cells (Figure 4A). Because we have previously shown that MED1 is highly phosphorylated and activated by HER2 pathway in BT474 cells, we further carried out to examine whether HER2 pathway may play a role in this unaffected MED1 promoter presence by fulvestrant. Interestingly, we found that treatment with HER2 inhibitor AG825 or ERK1/2 inhibitor PD98059 both dramatically inhibited the recruitment of MED1 to the *TFF1* promoter by fulvestrant in BT474 cells (Figure S3).

We then carried out further studies to examine the recruitments of MED1, ERα, RPB1 (RNA polymerase II subunit 1) on *TFF1* promoter after fulvestrant treatment alone, MED1 knockdown alone, or combined treatments of both using BT474 cells. We found that treatment with fulvestrant alone reduced the recruitments of ERα and RPB1 as expected. Interestingly, combined fulvestrant treatment with MED1 knockdown further decreased the recruitments of ERα, RBP1, and MED1 when compared to that of either treatment alone (Figure 4B). Histone deacetylation by histone deacetylases (HDACs, such as HDAC1), is an important mechanism in the transcriptional repression of ER and its target genes by antiestrogens [44,45]. Importantly, our recent studies found that MED1 can affect the recruitment of HDAC1 to TFF1 promoter in BT474 cells in response to another endocrine therapeutic agent Tamoxifen [40]. Therefore, we decided to perform additional ChIP experiments to investigate whether MED1 can affect the HDAC1 recruitment on TFF1 promoter by fulvestrant. As shown in Figure 4B, we observed an increased recruitment of transcriptional corepressor HDAC1 to *TFF1* promoter by this combined treatment. Additionally, we also conducted these experiments in ZR75-1 cells and obtained similar results (Figure S4). These results are consistent with the hypothesis that fulvestrant and MED1 regulate the expression of these ER target genes through modulating the recruitment of RNA polymerase II and transcriptional corepressor HDAC1.

### Knockdown of MED1 sensitizes xenograft tumors of breast cancer to Fulvestrant *in vivo*

**Figure 5:**
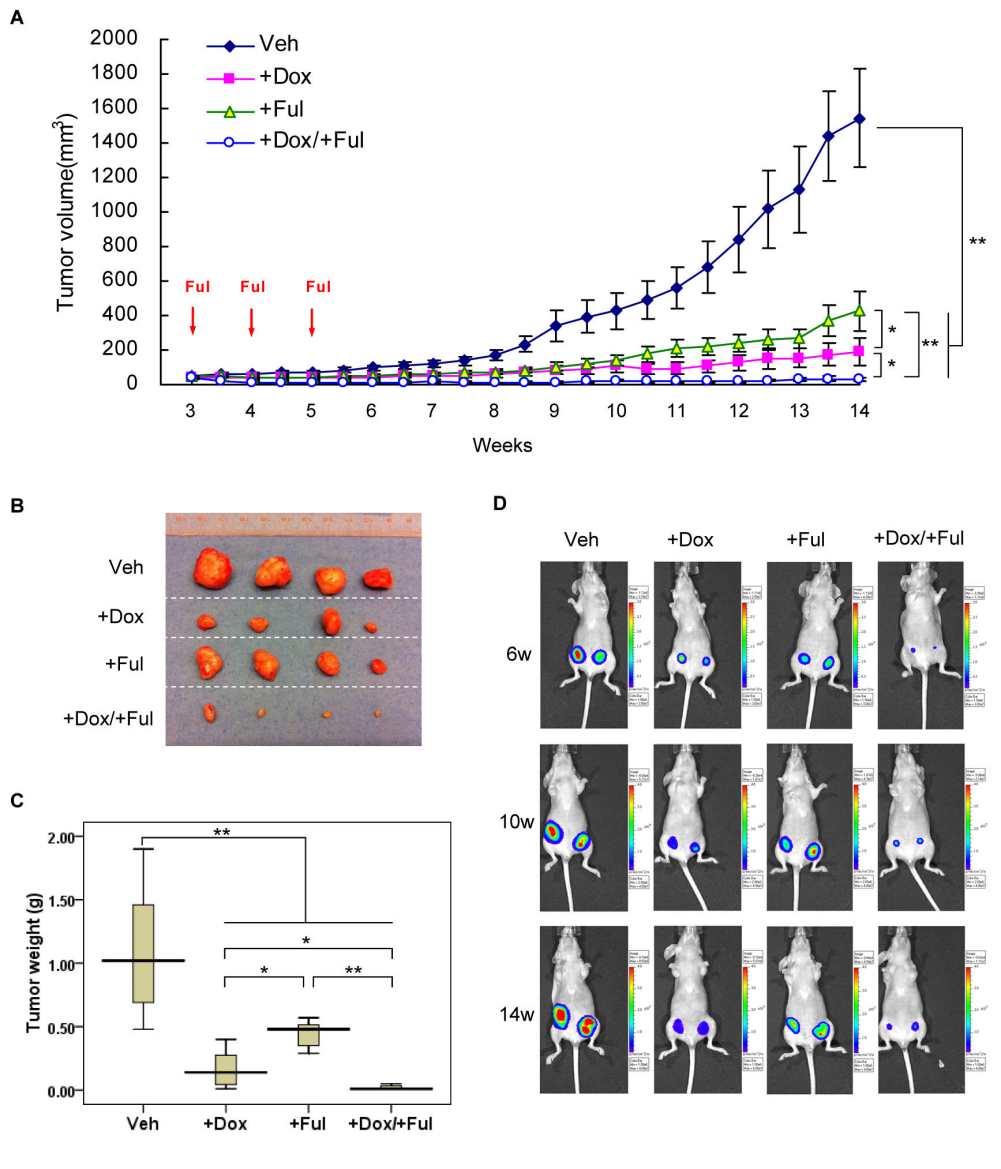
MED1 knockdown in combination with fulvestrant treatment dramatically inhibits the growth of orthotopic tumor xenografts. BT474-tet-shMED1 cells were injected into the fat pad of 5-6 weeks old nude mice. After 3 weeks, mice were randomly divided into four groups (4 mice/group): vehicle control (Veh), or doxycycline (+Dox), fulvestrant (+Ful) and the combination treatment (+Dox/+Ful) and examined for the followings: (**A**) Tumor volumes were measured twice per week by caliper (n=8). Red arrows indicate the time of fulvestrant treatments. (**B**) Tumors were harvested and the representative images of tumors in each group were shown. (**C**) Tumor weights were calculated and shown as a box-plot with median and whiskers from minimum to maximum (n=8). (**D**) Representative tumor bioluminescence images of each group at indicated time point were shown (*: P< 0.05; **: P< 0.01).

To determine the role of MED1 on the fulvestrant sensitivity *in vivo*, we further employed an orthotopic xenograft mouse model. BT474-tet-shMED1 cells were first orthotopically transplanted into the fat pad of nude mice. Tumors were then allowed to growth for 3 weeks to reach a size of approximately 50 mm^3^. These mice were then randomized into four treatment groups: vehicle control (Veh), doxycycline alone (+Dox), fulvestrant alone (+Ful), and combination (+Dox/+Ful). We found that both fulvestrant and Dox treatment alone significantly reduced the growth rate of the tumors compared with vehicle control group (Figure 5A). Importantly, combination of fulvestrant and doxycycline treatment further inhibited the tumor growth, with both tumor volumes and weights greatly regressed (Figure 5A, B and C). Similar phenomena were observed with bioluminescence detections of the expression of the integrated luciferase gene in these tumors (Figure 5D).

**Figure 6:**
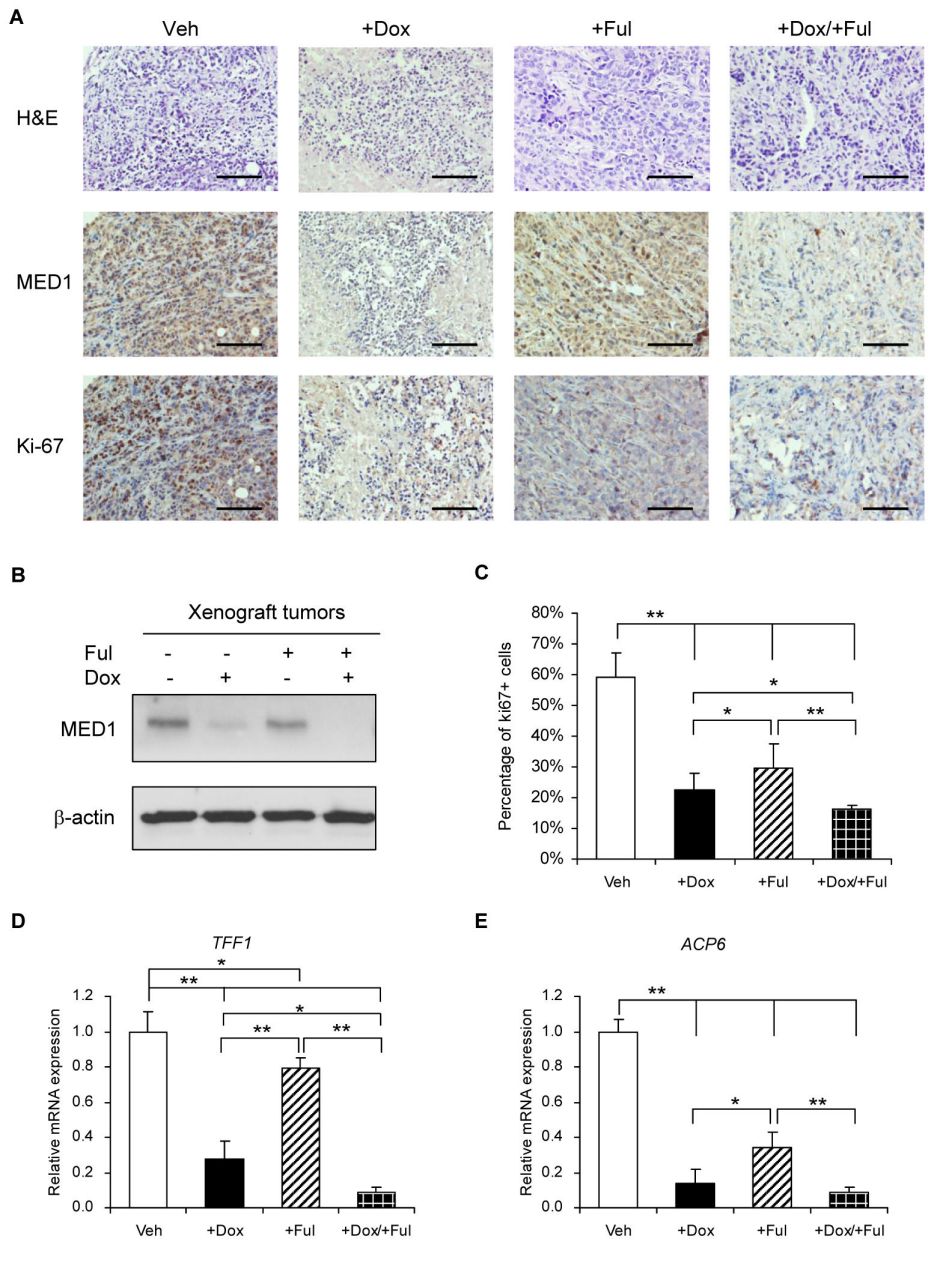
Knockdown of MED1 potentiates fulvestrant-repressed cell proliferation and ERα target genes’ expression in xenograft tumors. (**A**) Paraffin-embedded tissue sections of above xenograft tumors were subjected to H&E staining (top panels) and immunohistochemical (IHC) staining with antibodies against MED1 (middle panels) or Ki-67 (bottom panels). Scale bar = 100µm. (**B**) Western blot analyses of tumors using antibodies against MED1 and β-actin. (**C**) Quantification of Ki-67 positive cells in each group as shown in (A). (**D**) and (**E**) Total RNA of xenograft tumors from each group were extracted and analyzed for the mRNA expression of ERα target genes *TFF1* (D) and *ACP6* (E) by real-time RT-PCR. (n=6. *: P< 0.05; **: P< 0.01).

We further carried out IHC staining (Figure 6A) and western blot (Figure 6B) analyses and found that Dox treatment indeed successfully inhibited the expression of MED1 in these xenograft tumors. We also found that the percentage of tumor cells expressing the proliferation marker ki-67 was significantly reduced after fulvestrant or Dox treatment. Importantly, combined treatment with fulvestrant or Dox further reduced the number of ki-67 expressing tumor cells (Figure 6A and C). This result is consistent with our *in vitro* results showing that silencing MED1 potentiates the inhibitory effect of fulvestrant on cell proliferation *in vitro*. To further examine whether silencing MED1 also affects fulvestrant-repressed expression of ERα target genes in these xenograft tumors, semi-quantitative real time RT-PCR was performed on the total RNA isolated. As expected, we found that treatment with fulvestrant or Dox alone reduced the mRNA expression of both *TFF1* and *ACP6* genes in the xenograft tumors. Significantly, the combination of treatment with fulvestrant and Dox further decreased the mRNA expression of these genes when compared with treatment with either fulvestrant or Dox alone (Figure 6D and E).

## Discussion

After prolonged fulvestrant therapy, acquired resistance eventually occurs in most breast cancer patients, which has becoming a major clinical problem for human breast cancer treatment [7,9,10,14]. Through this study, we have provided evidences supporting a role for MED1 in fulvestrant resistance of breast cancer cells both *in vitro* and *in vivo*. We found that: 1) knockdown of MED1 sensitizes otherwise resistant BT474, ZR75-1 and MCF-7F cells to fulvestrant treatment; 2) MED1 knockdown works cooperatively with fulvestrant to inhibit the expression of endogenous ERα target genes and cell cycle progression; 3) knockdown of MED1 affects the recruitment of RNA polymerase II and transcriptional corepressor HDAC1 on the endogenous ER target gene promoter in the presence of fulvestrant; 4) down-regulation of MED1 in combination with fulvestrant dramatically inhibits the growth of orthotopic tumor xenografts *in vivo*.

As expected, fulvestrant is able to significantly reduce the recruitment of MED1 on the ER target gene promoter in fulvestrant sensitive MCF-7 cells. However, interestingly, we found that fulvestrant failed to affect the MED1 recruitment to the same target gene promoter in fulvestrant-resistant BT474 cells, despite a reduced ER recruitment (Figure 4B). We have previously shown that MED1 is highly phosphorylated and activated in BT474 cells, in which HER2 is known to be amplified and overexpressed, through HER2/ERK signaling pathway [40]. Therefore, one possibility is that once MED1 is activated by the HER2 pathway, it can still be highly recruited despite the reduced ER levels by fulvestrant treatment to activate ER target gene expression and thus confer fulvestrant resistance. Consistent with this, we found that fulvestrant treatment can now drastically reduce the MED1 recruitment after blocking the HER2 pathway with AG825 or PD98059. Furthermore, we found that MED1 is required for the expression of E2-induced ER target genes (e.g. *TFF1*, *Cyclin D1*) and those ER target genes (e.g. *ACP6, LIF*) activated by HER2 in the presence of fulvestrant ((Figure 3). Moreover, we found that knockdown of MED1 can further inhibit the expression of HER2 itself in the presence of fulvestrant (Figure S5). Taken together, these studies support a regulator loop between HER2 and MED1 in controlling fulvestrant resistance of human breast cancer cells.

Mechanistically, our data support a role of MED1 in mediating fulvestrant-regulated expression of these ER target genes potentially through collectively affecting promoter occupancy of RNA polymerase II, ER and transcriptional cofactors. Consistent with previous reports that MED1 exists predominantly in a Mediator subpopulation that enriches in RNA Polymerase II, we found knockdown of MED1 significantly blocked the recruitment of RPB1 (RNA polymerase subunit 1) to the ER target gene promoter [30]. Interestingly, we found ER occupancy on the *TFF1* promoter can also be further reduced by combined treatment of fulvestrant and MED1 knockdown. MED1 is a subunit of transcriptional coactivator Mediator complex that serves as a bridge between ER and RNA polymerase II general transcription machinery. Therefore, one possible reason for this is that the loss of MED1 causes ER to become unable to form a stable complex with RNA polymerase II and general transcription machinery on the target gene promoter, thus increasing the turnover and dissociation of ER from the promoter. Finally, we found that knockdown of MED1 led to increased promoter occupancy of transcriptional corepressor HDAC1. It has been previously proposed that the overall balance of coactivators and corepressors levels is an important determinant for their recruitment by ER in response to agonist or antagonist [44,46]. We reason that loss of MED1 could trigger a shifted equilibrium between corepressors and coactivators to favor the increased recruitment and promoter occupancy of corepressor such as HDAC1.

MED1 co-amplifies with HER2 and is overexpressed in about 40-60% of human breast cancer [32,36–38]. Recent studies have further shown that MED1 expression highly correlates with poor clinical outcome of breast cancer patients treated with endocrine therapy [39,47,48]. We have previously shown that MED1 is involved in tamoxifen resistance in human breast cancer [40]. In this study, we provided strong evidences for a key role of MED1 in the resistance to another class of endocrine therapy agent, fulvestrant, both *in vitro* and *in vivo*. Taken together, these studies demonstrated a broad role for MED1 in mediating endocrine resistance of human breast cancer cells, and support its potential usage as a possible therapeutic target to overcome endocrine resistance, alone or in combination with current endocrine therapy regiments. Interestingly, our recent studies revealed a previously unexpected tissue- and gene-specific role for MED1 *in vivo* in breast but not in other estrogen responsive tissue such as uterus and bone [31]. Targeting MED1 therefore may offer advantageous therapeutic outcome by overcoming both resistance and the severe adverse effects of currently used high dose regimens on other tissues.

## Materials and Methods

### Ethics statement

All of the animal experiments described in this study were approved by the Institutional Animal Care and Use Committee (IACUC) at the University of Cincinnati. All animals were maintained in accordance with IACUC guidelines.

### Cell culture and reagents

Human breast cancer cell lines BT474, ZR75-1 and MCF-7 cells were previously purchased from American type culture collection (ATCC). To generate these cells that express firefly luciferase (BT474-luc, ZR75-1-luc, MCF7-luc), the parents cells were transfected with EF1-luc plasmid and selected for clones stably expressing the firefly luciferase. pLKO-Tet-on-shMED1 plasmids were constructed by inserting shRNA sequences against MED1 or control GFP into pLKO-Tet-on vectors [49,50] and packaging them into lentiviruses as described [40]. These lentiviruses were then used to infect above luciferease expressing cell lines to generate BT474-tet-shMED1, ZR75-1-tet-shMED1 and MCF-7-tet-shMED1 and corresponding tet-shGFP control cells. All cells were maintained in DMEM medium supplemented with 10% fetal bovine serum (Hyclone) and 1µg/ml puromycin (Invitrogen). MCF7-F, a fulvestrant-resistant subline of MCF-7, was previously described [24]. These cell lines were further authenticated on the basis of viability, recovery, growth and morphology. The expression status of ERα and MED1 was further confirmed by Western blotting before they were used in the experiments. 17β-estradiol (E2) and fulvestrant(Ful, ICI 182,780) were purchased from Sigma. Doxycycline (Dox) was purchased from Fisher Scientific.

### Cell proliferation assay

MTT assays were performed to analyze the cell proliferation as described previously [40]. In brief, 5000 cells/well were cultured in 96-well plates and treated with indicated dose of fulvestrant for 7 days. Cells were incubated with 100µl/well of MTT (3-(4,5-Dimethyl-2-thiazolyl)-2,5-diphenyl-2H-tetrazolium bromide, Sigma) for 4 h. After removing the medium, 100µl/well of DMSO was added to each well and plates were shaken for 10 min. The absorbance of each well was measured at 570 nm using a Synergy II spectrophotometer (Biotek).

### Flow cytometric analysis

Cells were harvested by trypsinization, fixed in 70% ethanol and stained for total DNA content with PBS containing 50 µg/ml propidium iodide (Sigma) and 100 µg/ml RNase (Sigma) for 30 min at 37°C. The data of cell cycle distribution were collected using a FACS Calibur flow cytometer (BD Biosciences, San Diego, CA) and analyzed by the software FlowJo (Tree Star, Ashland, OR).

### Western blot analysis

Whole cell extract were obtained by treating cells with RIPA buffer (50mM Tris pH 8.0, 150mM NaCl, 1% Triton X-100, 0.5% Sodium deoxycholate, 0.1% SDS, 2mM EDTA, 5% Glycerol, protease inhibitors cocktail(Roche), 0.1M DTT and 0.1M PMSF) for 30 min on ice. 50µg of each extract, as measured by standard Bradford assay, were subjected to SDS-PAGE and western blotting analysis using Anti-MED1 antibody [30] and anti-β-actin (A2066, Sigma) antibodies.

### Quantitative real time PCR

Total RNA was isolated using RNeasy Mini Kit (Qiagen). The first-strand cDNA was synthesized by reverse transcription of mRNA using oligo(dT) 20 primer and SuperScript^TM^ III Reverse Transcriptase (Invitrogen). Real time PCR was performed with SYBR Green PCR Master Mix reagents (Roche) using ABI Prism 7900 HT (Applied Biosystems). Primer sequences for *TFF1, CyclinD1, LIF, ACP6* and *GAPDH* were previously described [40]. Relative gene expression was analyzed using the 2^-ΔΔCT^ method by normalization to the *GAPDH* levels [51].

### Chromatin immunoprecipitation (ChIP)

ChIP assays were performed as previously described [40,51]. In brief, after indicated treatments, cells were fixed using 1% formaldehyde for 10 min. After PBS washing, cells were harvested, lysed and sonicated to generate an average DNA size of 0.2-1 kb using a Bioruptor (Diagenode). Immunoprecipitations were then carried out using control pre-immume IgG and antibodies against MED1 [30], ERα (HC-20, Santa Cruz), HDAC1(C-19, Santa Cruz) and RPB1 [30]. Real-time PCR amplifications were performed after reverse cross-linking and extraction of DNA from immunoprecipitated chromatin. The primers for *TFF1* promoter were described previously [40].

### Orthotopic xenograft assay

5-6 weeks old female nude mice (01B74-Athymic NCr-nu/nu) were ordered from NCI, Frederick. 2×10^6^ BT474-tet-shMED1 cells were suspended in 50% Matrigel (BD Bioscience) and injected into the fat pad of the fourth glands. Vehicle or 2 mg/ml Dox were supplemented in daily drinking water containing 2.5% sucrose. When tumors reached the size of ~50 mm^3^, the mice were further randomly distributed into four groups with 4 mice each and treated with control vehicle, doxycycline (+Dox), fulvestrant (+Ful), or both (+Dox/+Ful). 5 mg of fulvestrant were dissolved in 100% ethanol and diluted in olive oil before being injected subcutaneously once per week for 3 weeks. Tumors were measured with calipers twice a week and the tumor volumes were calculated (Volume (mm^3^) = π×length×width^2^ /6). Tumor progression was also monitored periodically by bioluminescence imaging using IVIS^®^ Lumina Living Image System (Caliper). At the end of study, tumors were harvested, fixed, and embedded in paraffin. Tumor sections were subjected to standard H&E staining and immunohistochemical (IHC) staining using anti-MED1 [30] and anti-Ki67 (Ab-4, NeoMarkers) antibodies [31].

### Statistical analysis

All data were expressed as mean ± SD and two-tailed Student’s *t*-tests were used for analyzing statistical differences between two groups. For the assays including more than two groups, Dunn’s *post hoc* t-test was used to compare all of each groups following one-way ANOVA. Statistical significance was defined as: *p<0.05, **p<0.01.

## Supporting Information

Figure S1 Silencing MED1 sensitizes BT474 and ZR75-1 cells to fulvestrant. (TIF)

Figure S2 Silencing MED1 inhibited ERE-TFF1-Luc reporter gene in MCF7-F cells. (TIF)

Figure S3 HER2 and ERK1/2 inhibitors block MED1 recruitment on *TFF1* promoter in BT474 cells in the presence of fulvestrant. (TIF)

Figure S4 MED1 affects the recruitment of RNA pol II and transcriptional corepressor HDAC1 to *TFF1* promoter by fulvestrant in ZR75-1 cells. (TIF)

Figure S5 MED1 knockdown impairs the expression of HER2 gene in the presence of fulvestrant. (TIF)
