# Supplementary figures and images for: Silencing MED1 Sensitizes Breast Cancer Cells to Pure Anti-Estrogen Fulvestrant In Vitro and In Vivo
Source: PLoS One. 2013 Jul 30;8(7):e70641. doi: 10.1371/journal.pone.0070641 (PMC3728322; doi:10.1371/journal.pone.0070641)

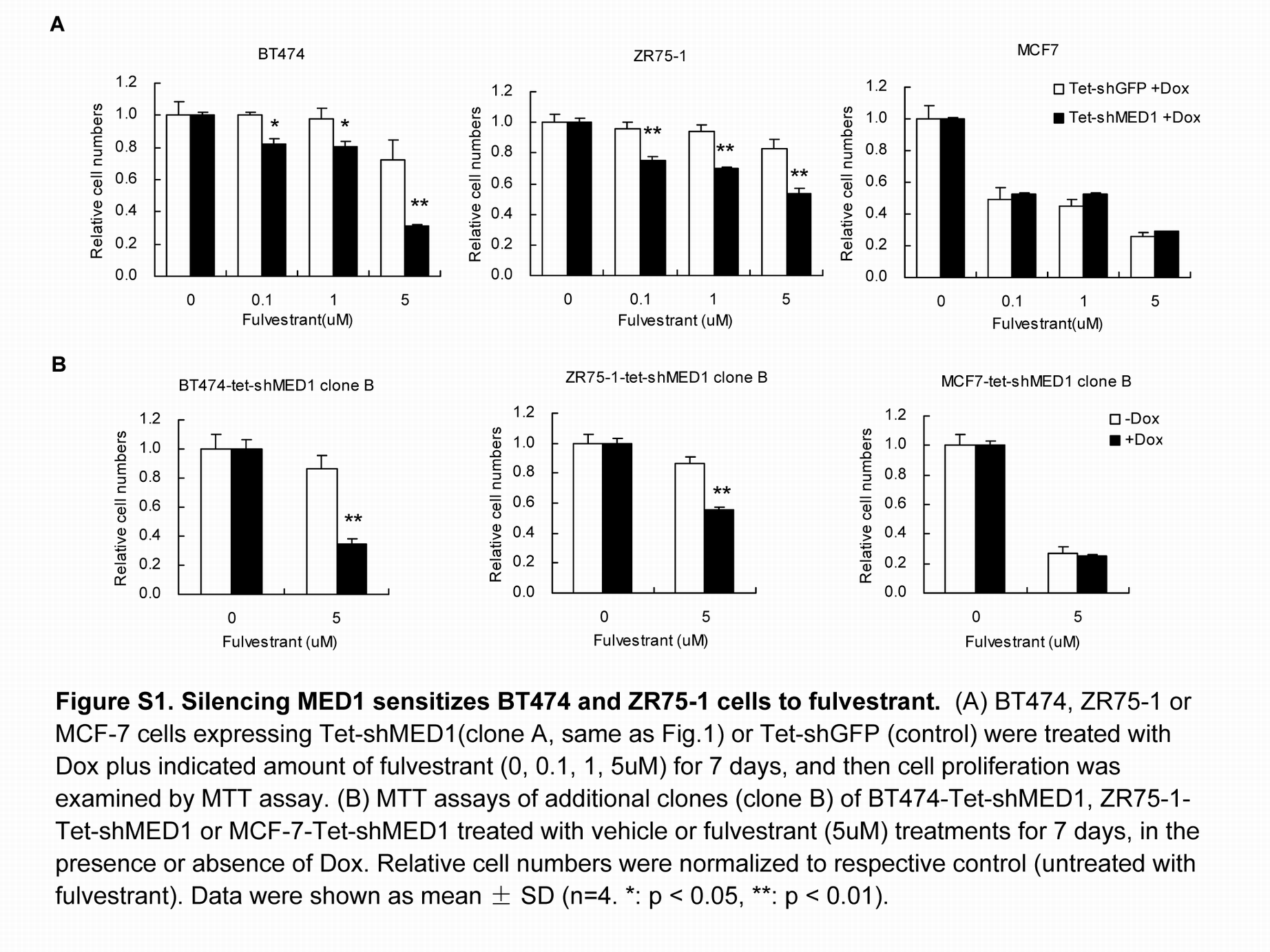

Supplement: Figure S1 — (TIF) [file pone.0070641.s001.tif]

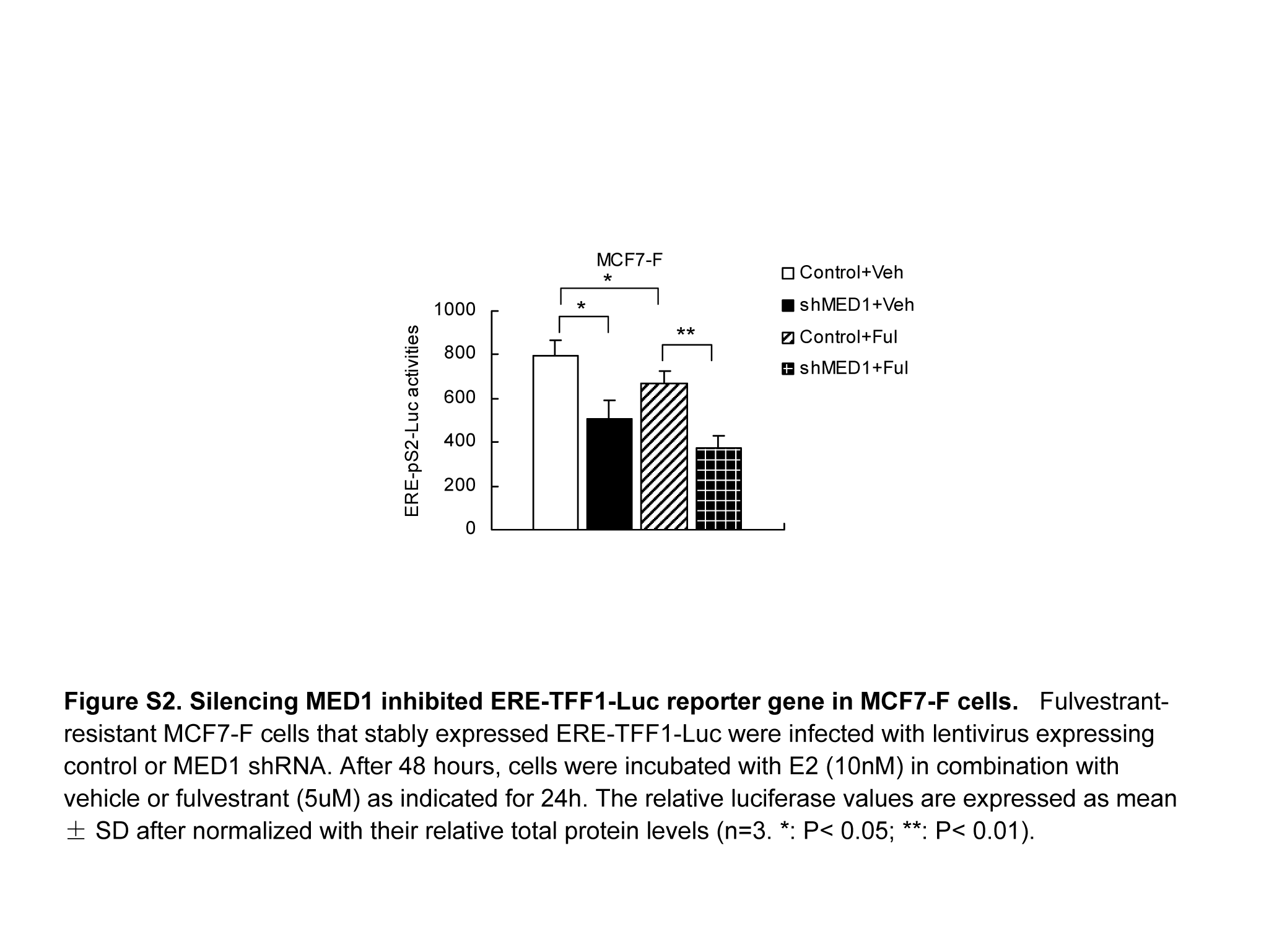

Supplement: Figure S2 — (TIF) [file pone.0070641.s002.tif]

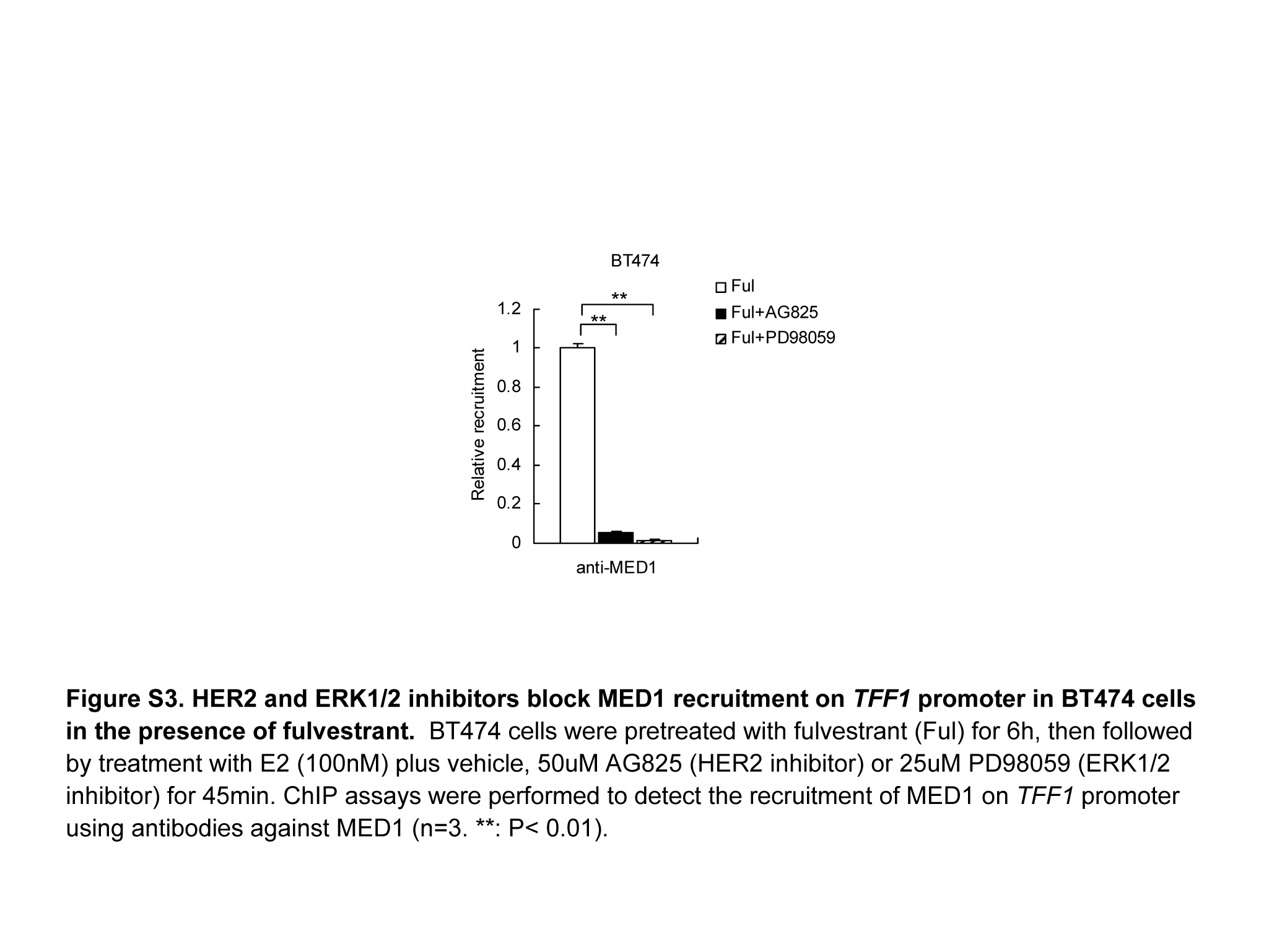

Supplement: Figure S3 — (TIF) [file pone.0070641.s003.tif]

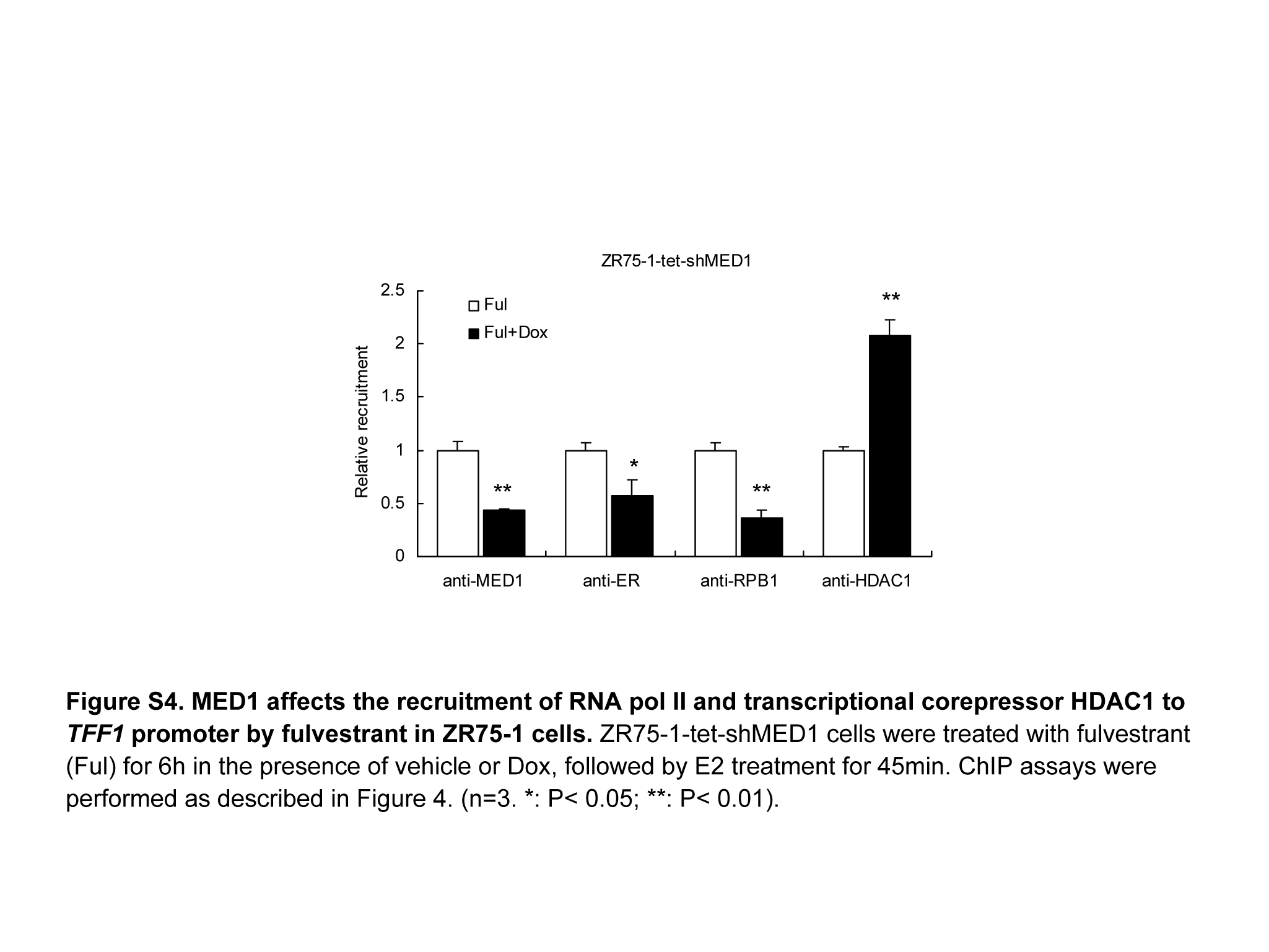

Supplement: Figure S4 — (TIF) [file pone.0070641.s004.tif]

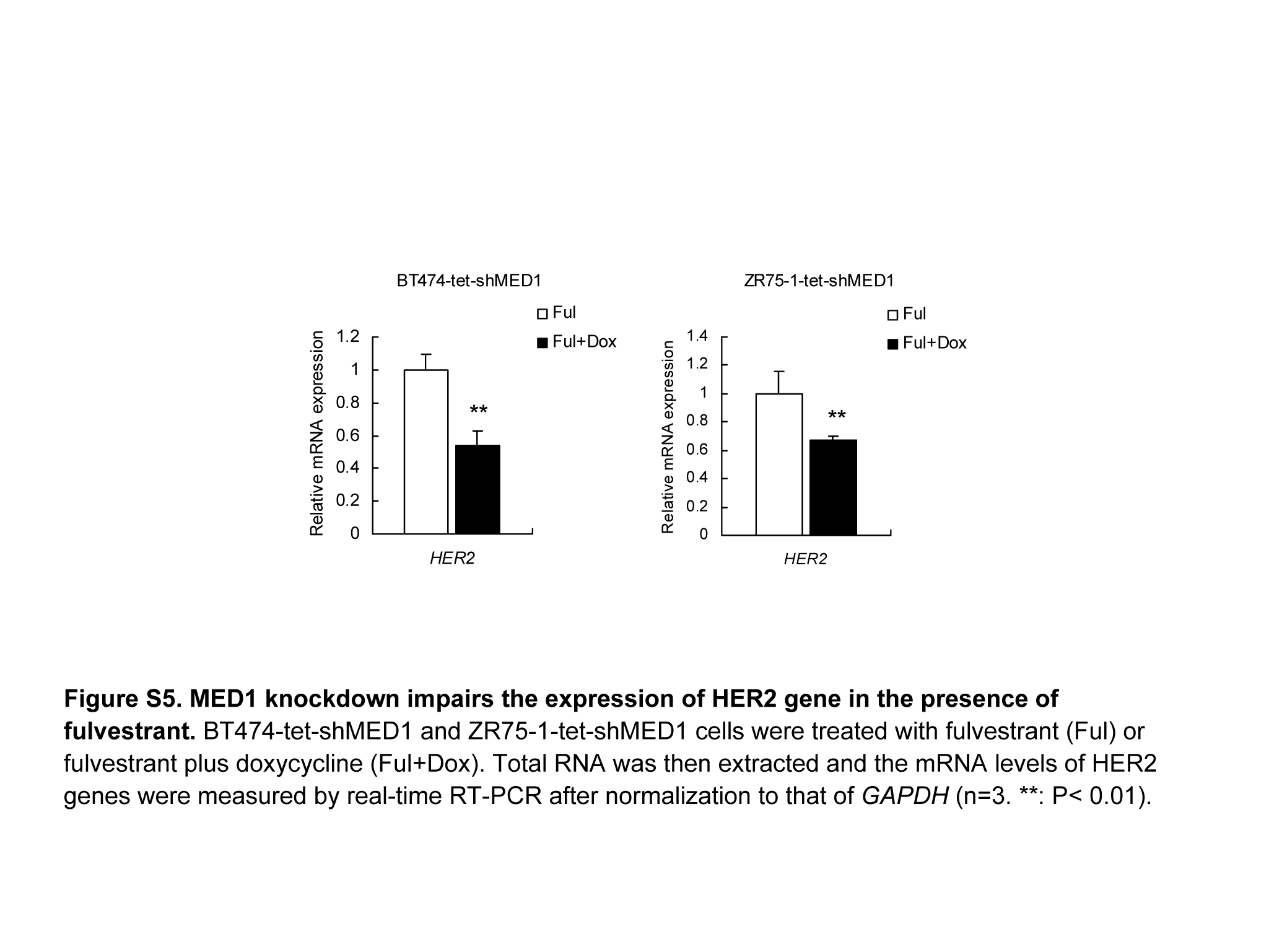

Supplement: Figure S5 — (TIF) [file pone.0070641.s005.tif]
